# S-Allyl-L-Cysteine Affects Cell Proliferation and Expression of H_2_S-Synthetizing Enzymes in MCF-7 and MDA-MB-231 Adenocarcinoma Cell Lines

**DOI:** 10.3390/biom14020188

**Published:** 2024-02-04

**Authors:** Anna Bentke-Imiolek, Dominika Szlęzak, Marta Zarzycka, Maria Wróbel, Patrycja Bronowicka-Adamska

**Affiliations:** Jagiellonian University Medical College, Faculty of Medicine, Chair of Medical Biochemistry, 7 Kopernika Street, 31-034 Kraków, Poland; dominika.szlezak@uj.edu.pl (D.S.); marta.zarzycka@uj.edu.pl (M.Z.); mtk.wrobel@uj.edu.pl (M.W.); patrycja.bronowicka-adamska@uj.edu.pl (P.B.-A.)

**Keywords:** S-allyl-L-cysteine (SAC), breast adenocarcinoma, 3-mercaptopyruvate sulfurtransferase (MPST), cystathionine γ-lyase (CTH), cystathionine β-synthase (CBS)

## Abstract

S-allyl-L-cysteine (SAC) is a sulfur compound present in fresh garlic. The reference literature describes its anticancer, antioxidant and neuroprotective effects. Breast cancer is infamously known as one of the most commonly diagnosed malignancies among women worldwide. Its morbidity and mortality make it reasonable to complete and expand knowledge on this cancer’s characteristics. Hydrogen sulfide (H_2_S) and its naturally occurring donors are well-known investigation subjects for diverse therapeutic purposes. This study was conducted to investigate the SAC antiproliferative potential and effect on three enzymes involved in H_2_S metabolism: 3-mercaptopyruvate sulfurtransferase (MPST), cystathionine γ-lyase (CTH), and cystathionine β-synthase (CBS). We chose the in vitro cellular model of human breast adenocarcinomas: MCF-7 and MDA-MB-231. The expression of enzymes after 2, 4, 6, 8, and 24 h incubation with 2.24 mM, 3.37 mM, and 4.50 mM SAC concentrations was examined. The number of living cells was determined by the MTS assay. Changes in cellular plasma membrane integrity were measured by the LDH test. Expression changes at the protein level were analyzed using Western blot. A significant decrease in viable cells was registered for MCF-7 cells after all incubation times upon 4.50 mM SAC exposure, and after 6 and 24 h only in MDA-MB-231 upon 4.50 mM SAC. In both cell lines, the MPST gene expression significantly increased after the 24 h incubation with 4.50 mM SAC. S-allyl-L-cysteine had opposite effects on changes in CTH and CBS expression in both cell lines. In our research model, we confirmed the antiproliferative potential of SAC and concluded that our studies provided current information about the increase in MPST gene expression mediated by S-allyl-L-cysteine in the adenocarcinoma in vitro cellular model for the MCF-7 and MDA-MB-231 cell lines. Further investigation of this in vitro model can bring useful information regarding sulfur enzyme metabolism of breast adenocarcinoma and regulating its activity and expression (gene silencing) in anticancer therapy.

## 1. Introduction

Breast cancer is infamously known as one of the most commonly diagnosed malignancies among women worldwide, accounting for as much as 36% of oncological patients. It is estimated that 2.1 to 2.3 million new cases of breast cancer are diagnosed globally each year. In Poland, it is also the most commonly diagnosed malignant tumor in women, with a steady increase in reported diagnosis. It is also still the major cause of death in overall cancer cases. The percentage of five-year survival due to breast cancer in Poland is 78.5%, differing significantly from, for example, the result of 90% achieved in the United States. The epidemiological data and present severity of breast cancer morbidity and mortality make it reasonable to seek research-based information to complete and expand knowledge on the characteristics of this cancer [1,2].

The MDA-MB-231 is an epithelial, human breast cancer cell line that was developed from a pleural effusion of a 51-year-old Caucasian female with a metastatic mammary adenocarcinoma [3]. It is characterized by its invasiveness, highly aggressive growth and poorly differentiated triple-negative features. It lacks epidermal growth factor receptor 2 (HER) amplification as well as estrogen and progesterone receptor expression. Thus, it leaves human breast cancer with very limited treatment options [4,5]. The MCF-7 cell line was developed from a pleural effusion, of a 69-year-old woman diagnosed with a breast adenocarcinoma [6]. Contrary to MDA-MB-231, it is characterized by the presence of estrogen, progesterone, and glucocorticoid receptors. It is a potent model for a variety of basic research [7,8].

**Figure 1:**
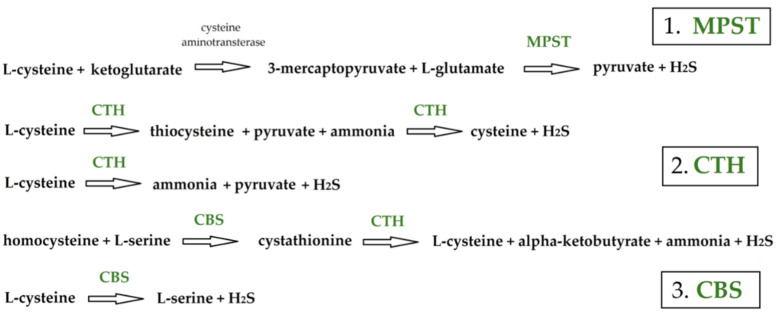
Transformation of sulfur-containing compounds as a part of H_2_S synthesis with the involvement of 3-mercaptopyruvate sulfurtransferase (MPST), cystathionine γ-lyase (CTH), and cystathionine β-synthase (CBS) enzymes.

Following a plant-based diet is described to be of great importance for breast cancer primary prevention. It is considered a valuable source of natural bioactive compounds, involving both bioactive nutrients and non-nutrients, such as sulfur compounds. Thus, the presented study is consistent with studies on the chemopreventive and chemosensitizing effects of the bioactive compounds in the plant-based diet for breast cancer [9]. Hydrogen sulfide (H_2_S) and its naturally occurring donors are well-known investigation subjects for diverse therapeutic purposes [10]. Among well described abilities of this group of compounds, proliferation inhibition is one of the commonly investigated features [11]. The class of compounds called isothiocyanates is a potentially rich source of H_2_S donors. They are secondary plant metabolites, products of glucosinolates hydrolysis, found exclusively in cruciferous vegetables—the family *Brassicaceae* [12]. Isothiocyanates have been described in the literature to demonstrate antiproliferative, pro-apoptotic, anti-inflammatory, antimigratory, and antiangiogenic effects against several cancers. The ability to modulate the redox environment and signaling pathways as well as regulate the expression and activity of DNA methyltransferases, histone modifiers, and miRNA was described among others [13,14]. The phytotherapeutic and nutraceutic usefulness of *Brassicaceae* in the prevention of human diseases, such as cancer, neurodegenerative processes, and cardiovascular diseases, has been widely discussed in the literature. Although these effects have been associated with isothiocyanates, the exact mechanism of action is still unknown [11]. A very well described feature of naturally occurring sulfur compounds is their ability to inhibit proliferation. Garlic-derived compounds known for their antiproliferative abilities are S-propyl-L-cysteine (SPC) and S-propargyl-L-cysteine (SPRC) [15]. S-allyl-L-cysteine (SAC) is a promising organosulfur compound exhibiting a considerable number of positive actions in cell models and living systems. SAC is a compound naturally derived from fresh garlic (*Allium sativum*) and concentrated in fermented black garlic. The research data presented its anticancer, antihepatotoxic, neuroprotective, neurotrophic activity, and antioxidative activity in cellular and animal models [16,17]. S-allyl-L-cysteine was described to induce cytotoxic effects in human lung cancer cell lines via induction of oxidative damage, downregulation of Nrf2, NF-κB and apoptosis [18]. It was also suggested that SAC is a potential source of H_2_S and is responsible for the cardioprotective effects of garlic [15]. The search for the presence and role of enzymes involved in sulfur metabolism within cancer cells is a commonly undertaken topic in recent years but still not fully described [19,20]. The enzymes 3-mercaptopyruvate sulfurtransferase (MPST), cystathionine γ-lyase (CTH), and cystathionine β-synthase (CBS) were included in the present investigation. MPST is a multifunctional enzyme, and it belongs to the class of enzymes called sulfurtransferases that transfer sulfur atoms and are involved in H_2_S endogenous formation from L-cysteine. The second enzyme CTH is also a sulfurtransferase and pyridoxal phosphate (PLP)-dependent enzyme involved in H_2_S production and sulfur-containing compound metabolism. The third enzyme, similar to CTH by its PLP dependence, is CBS (Figure 1) [19,20]. Endogenously produced hydrogen sulfide may occur in human tissues through enzymatic and non-enzymatic pathways, enzymatic being considered primary in generating H_2_S. Non-enzymatic H_2_S generation involves thiol or thiol-containing compound reactions, e.g., the reduction of dietary inorganic polysulfides by reduced glutathione (GSH) or the hydrolysis of inorganic sulfide salts leading to non-enzymatic H_2_S generation [21,22]. S-allyl-cysteine was described as an endogenous H_2_S donor that ameliorates carbon tetrachloride-induced liver fibrosis in rats through antioxidant, anti-inflammatory and antifibrotic effects [23]. It was also described that sulfide stress is characterized by an increase in H_2_S/polysulfide production as a result of elevated levels of MPST enzyme [24]. 

The MCF-7 cell line is a common research model that responds well to antiapoptotic, antiproliferative and other anticancer stimuli, while the triple-negative MDA-MB-231 line is widely described as resistant to therapeutic agents in which it is difficult to induce a corresponding effect. The null hypothesis of the presented study was that the SAC would effectively decrease cancer cell numbers and viability upon increasing SAC concentrations in the MCF-7 cell line and that the sulfur enzyme expression levels may be changed by its presence. We anticipate observing similar effects for the MDA-MB-231 cell to the MCF-7 cell line but foresee them to be much less potent in terms of the results achieved.

## 2. Materials and Methods

### 2.1. Cell Cultures

The MCF-7 breast adenocarcinoma cell line was derived from American Type Culture Collection (ATCC, Manassas, VA, USA). The adenocarcinoma cell line—MDA-MB-231—was a gift from the Department of Glycoconjugate Biochemistry (Jagiellonian University, Faculty of Biology, Institute of Zoology and Biomedical Research). Cells were cultured in a monolayer in the DMEM medium containing 10% fetal bovine serum (both: Biowest, Nuaillé, France) and with 100 U/mL penicillin and 100 µg/mL streptomycin solution (Thermo Fisher Scientific, Waltham, MA, USA). Additionally, the MCF-7 culture medium was supplemented with 10 μg/mL insulin (Sigma, Saint Louis, MO, USA). Cells were passaged regularly in a sterile chamber with laminar airflow, dissociated from plastic culture dishes with pre-warm 0.25% trypsin solution (Sigma, Saint Louis, MO, USA). Cells were counted in the Bürker chamber and seeded onto 100 mm diameter dishes (1.0 × 10^6^ cells/10 mL culture medium), 6-multi-well plate (0.15 × 10^6^ cells/1.5 mL culture medium) or 96-multi-well plate (0.012 × 10^6^ cells/0.15 mL culture medium) according to the experimental needs.

After 24 h, the medium was changed and cells were incubated in the absence or presence of three tested SAC concentrations: 2.24 mM, 3.37 mM, and 4.50 mM. SAC is known as a water-soluble bioactive compound [16]; as such, it was added directly to the medium solution. The concentration panel was chosen after careful literature search and taking into consideration our previous research regarding SAC apoptosis induction in the MCF-7 cellular model, where we established cytotoxicity and half maximal inhibitory concentration for 24 h and 48 h incubation [25]. As the initial tested concentration, one being estimated as the IC50 for 24 h incubation, that equal to 2.24 mM was taken into consideration. The dosage was doubled in final tested concentration and incubation times were set from 2 h to a maximum of 24 h. All solutions were pre-warmed to 37 °C to meet the temperature of the culture medium. Each experiment was accompanied by a control with complete culture medium alone added to the cells.

### 2.2. Cell Membrane Integrity by the LDH Test

The colorimetric LDH (lactate dehydrogenase) cytotoxicity assay—Cytotoxicity Detection Kit (Thermo Fisher Scientific, Waltham, MA, USA)—was used to assess the cytotoxicity of SAC towards the cell, indicated by the presence of LDH in the medium. The principle of the assay is based on the colorimetric measurement of lactate dehydrogenase activity. Lactate dehydrogenase is a cytosolic enzyme, not released into the environment under physiological conditions. As a result of mechanical rupture of the plasma membrane, LDH leaks out into the culture medium. This initiates enzymatic reactions with the test substrate branching to produce the colored product. The increase in LDH activity in the culture medium directly correlates with the amount of colored product formed during incubation [26]. The cells cultured in 96-well plates were tested after 2, 4, 6, 8, and 24 h incubation with SAC concentrations of 2.24 mM, 3.37 mM, and 4.50 mM. The cytotoxicity assay was conducted according to the manufacturer’s protocol (test controls with Triton X and a clear culture medium). Experiments were performed at least 3 times in 4 to 6 technical repetitions. Spectrophotometric measurement was carried out in an Epoch Microplate BIO-TEK pellet reader (BIO-TEK Instruments Inc., Winooski, VT, USA).

### 2.3. Cell Viability by the MTS Test

For the determination of the amount of living cells, the commercial MTS assay was used (Promega, Madison, WI, USA). This colorimetric method uses the reduction of MTS (phenazine methosulfate) to measure cellular metabolic activity as a proxy for cell viability. Viable cells contain NADPH-dependent oxidoreductase enzymes, which reduce the MTS reagent to a colored product, formazan. Only living cells have the ability to produce formazan, which enables quickly and accurately determining the percentage of functioning cells and the effect of the tested factor on the viability of any cell line [27,28]. The cells cultured in 96-well plates were tested after 2, 4, 6, 8, and 24 h incubations with SAC concentrations of 2.24 mM, 3.37 mM, and 4.50 mM. Following, the MTS assay was conducted according to the manufacturer’s protocol, and the incubation lasted for 2 h. Experiments were performed at least 3 times in 4 to 6 technical repetitions. Spectrophotometric measurement was carried out in an Epoch Microplate BIO-TEK pellet reader (BIO-TEK Instruments Inc., Winooski, VT, USA).

### 2.4. Gene Expression at the Protein Level by Western Blot Analysis

(a) Protein extraction and concentration determination: The cells were treated with RIPA lysis buffer with a proteinase of inhibitor cocktail (10 mM Tris-HCl, pH 8; 1 mM EDTA, 0.1% SDS, 150 mM NaCl), then sonicated and centrifuged at 14,000× *g* for 15 min. The protein concentration was determined by the Bradford method [29]. Each sample was tested in 6 repetitions on a 96-well plate and analyzed in BIO-TEK apparatus.

(b) SDS-PAGE electrophoresis: Electrophoresis was carried out in Laemmli’s buffer system on 4% concentration gel and on 10% separation polyacrylamide gel in the presence of SDS [30]. The amount of protein in the samples was determined beforehand and an equal amount of each sample—10 µg per well—was applied. Bio-Rad’s Mini-PROTEAN Tetra Cell apparatus was used.

(c) Electrotransfer with chemiluminescence identification: Electrotransfer on the PVDF (polyvinylidene fluoride) membrane was conducted according to the Bio-Rad protocol on the Mini-PROTEAN Tetra Cell system (overnight at 4 °C; 0.15 A). All membranes were cut into parts taking into consideration the molecular weight of the investigated proteins (molecular weight: MPST 33 kDa, CTH 40–45 kDa, and CBS 63 kDa) and in consideration of the protein ladder added on the first and last wells. The relative amounts of investigated enzymes were determined using the appropriate primary antibody: anti-MPST (1:1000), anti-CTH (1:1000), and anti-CBS (1:1000) (Cell Signaling, Danvers, MA, USA). Anti-β-actin (1:5000, Abnova, Taipei, Taiwan) and anti-GAPDH (glyceraldehyde-3-phosphate dehydrogenase) (1:1000, Cell Signaling, Danvers, MA, USA) antibody were used to control equal loading. Prober secondary antibody was used: HRP-conjugated goat anti-mouse IgG antibody (1:10,000) or HRP-conjugated goat anti-rabbit IgG antibody (1:10,000) (Cell Signaling, Danvers, MA, USA). Proteins were visualized by chemiluminescence with SignalFire™ Elite ECL Reagent (Cell Signaling, Danvers, MA, USA). Each experiment was repeated a minimum of 3 times, with a minimum of two WB runs with similar results.

(d) Densitometric Evaluation of Western blot bands: The photos saved in the jpeg format (ChemiDoc MP^TM^ Imaging System with Image Lab Software, version 6.0, Bio-Rad, Hercules, CA, USA) were used for densitometry analysis. To obtain quantitative results, immunoblots were scanned using the public domain ImageJ software (National Institute of Health, Bethesda, MD, USA, http://rsbweb.nih.gov/ij/). Each data point was normalized against its corresponding β-actin/GAPDH data point. The error bars represent the standard deviation from three Western blot runs from each experiment performed.

### 2.5. Statistical Analysis

All results were represented as arithmetic means with standard deviations (SD). The normal distribution of data was verified by the Shapiro–Wilk test. Levene’s test was used for assessing homogeneity of variance. Statistical analysis was performed via the non-parametric Kruskal–Wallis test with the post hoc multiple comparison by Conover–Inman’s test (MTS test) and via the one-way analysis of variance (ANOVA), followed by Dunnett’s post hoc comparison test (Western blot analysis) using StatSoft Statistica 13 software (Tibco, Palo Alto, CA, USA). Differences with a *p* value < 0.05 were considered statistically significant. * *p* < 0.05, ** *p* < 0.01, and *** *p* < 0.001. Original Western blot pictures can be found in Supplementary Materials.

## 3. Results

### 3.1. Cell Membrane Integrity and Viability

The obtained data indicated no cytotoxic effect of SAC on the MCF-7 and MDA-MB-231 adenocarcinoma cell lines. For both lines tested, no LDH activity was observed in selected time intervals and at selected factor concentrations. The only exception was the value obtained for MCF-7 cells incubated 24 h with a SAC concentration of 2.24 mM; however, it did not exceed 1%, which confirms the lack of cytotoxic effect of the tested compound. The data are presented in Supplementary Materials (Tables S1 and S2).

**Figure 2:**
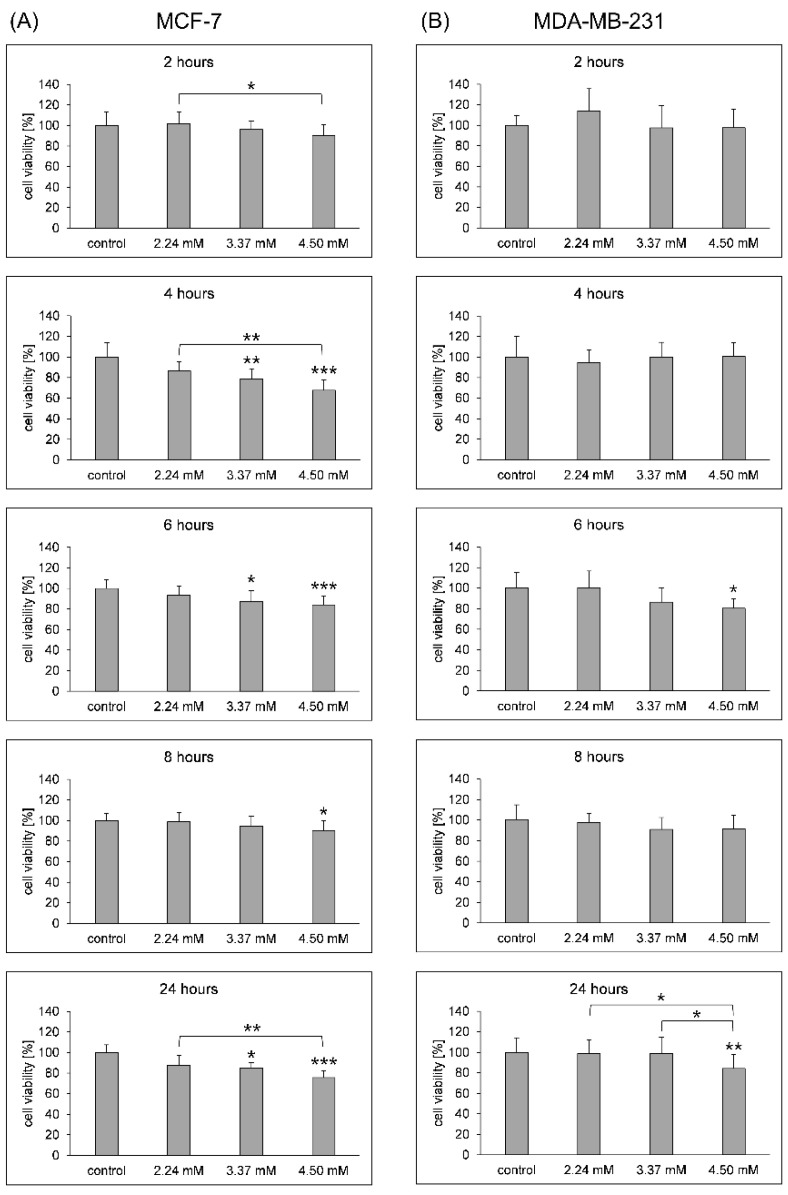
Cell viability by the MTS test. (**A**) MCF-7 adenocarcinoma cells after 2, 4, 6, 8, and 24 h incubation with 2.24 mM, 3.37 mM, and 4.50 mM SAC concentrations, (**B**) MDA-MB-231 adenocarcinoma cells after 2, 4, 6, 8 and 24 h incubation with 2.24 mM, 3.37 mM, and 4.50 mM SAC concentrations. * *p* < 0.05, ** *p* < 0.01, and *** *p* < 0.001 SAC-treated cells vs. control (unless otherwise stated).

A statistically significant decrease in cell viability was observed in the MCF-7 cell line with different concentrations of SAC and incubation times. Two-hour incubation with 4.50 mM SAC reduced the number of living cells by 10% (Figure 2A). Increasing the incubation time to 4 h with the investigated compound resulted in a registration of viability values of 86%, 79% and 68% for increasing SAC concentrations. After 6 h of incubation with 3.37 mM and 4.50 mM, we observed a decrease in cell viability of approximately 13% and 16%, respectively, and a viability reduced by 10% for a concentration of 4.50 mM after 8 h of incubation with SAC. The longest 24 h incubation of MCF-7 cells with SAC resulted in a reduction in the number of viable cells by 13%, 15%, and 25% for increasing agent concentrations: 2.24 mM, 3.37 mM, and 4.50 mM, respectively (Figure 2A).

The decreased number of viable cells in the MDA-MB-231 cell line was observed in two registered incubation times at statistically significant level for the higher SAC concentration used (Figure 2B). Six-hour incubation with 4.50 mM SAC reduced the number of living cells by 20%. The longest 24 h incubation resulted in viable cells at the level of 84% for 4.50 mM SAC. Two lower tested SAC concentrations did not affect the cell viability in this cell line.

### 3.2. Gene Expression by Western Blot Analysis

**Figure 3:**
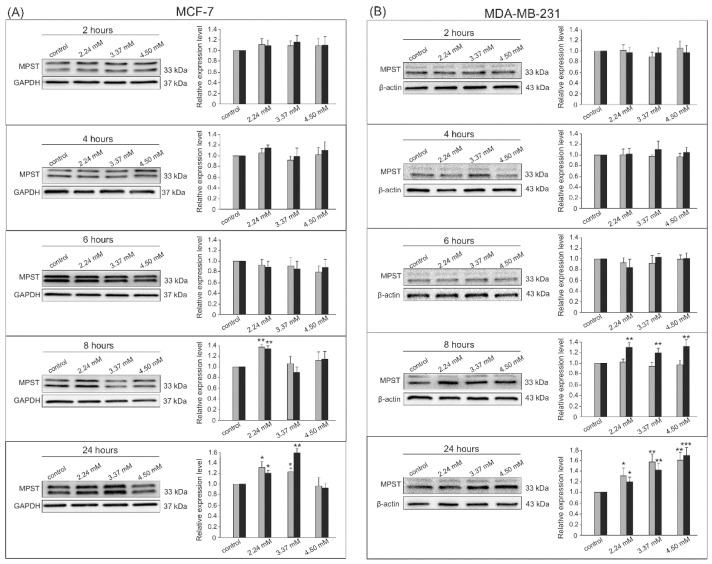
MPST expression by Western blot analysis with chemiluminescent detection and densitometric evaluation of the bands. The lower MPST band of 33 kDa is represented by black bar on the graph (on the right; the grey one representing the upper band). (**A**) MCF-7 adenocarcinoma cells after 2, 4, 6, 8, and 24 h incubation with 2.24 mM, 3.37 mM, and 4.50 mM SAC concentrations, (**B**) MDA-MB-231 adenocarcinoma cells after 2, 4, 6, 8, and 24 h incubation with 2.24 mM, 3.37 mM, and 4.50 mM SAC concentrations. * *p* < 0.05, ** *p* < 0.01, and *** *p* < 0.001 SAC-treated cells vs. control. Results show representative Western blot images and densitometric evaluation of the bands from all experiments.

**Figure 4:**
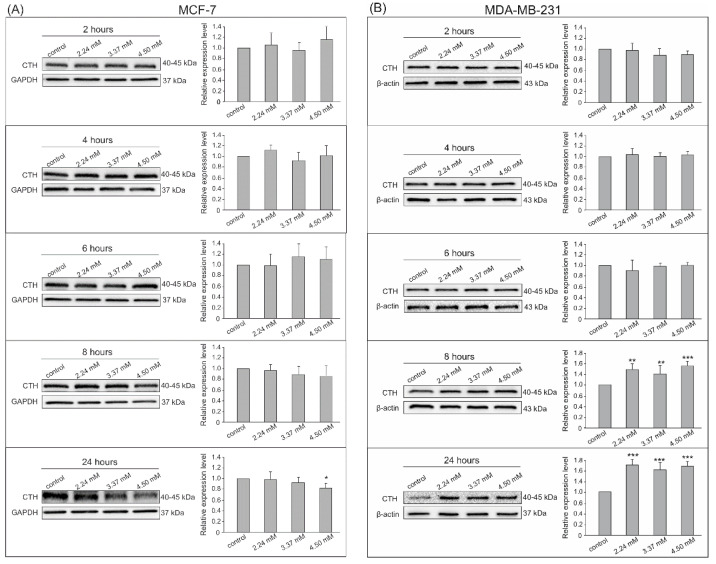
CTH expression by Western blot analysis with chemiluminescent detection and densitometric evaluation of the bands. (**A**) MCF-7 adenocarcinoma cells after 2, 4, 6, 8, and 24 h incubation with 2.24 mM, 3.37 mM, and 4.50 mM SAC concentrations, (**B**) MDA-MB-231 adenocarcinoma cells after 2, 4, 6, 8, and 24 h incubation with 2.24 mM, 3.37 mM, and 4.50 mM SAC concentrations. * *p* < 0.05, ** *p* < 0.01, and *** *p* < 0.001 SAC-treated cells vs. control. Results show representative Western blot images and densitometric evaluation of the bands from all experiments.

**Figure 5:**
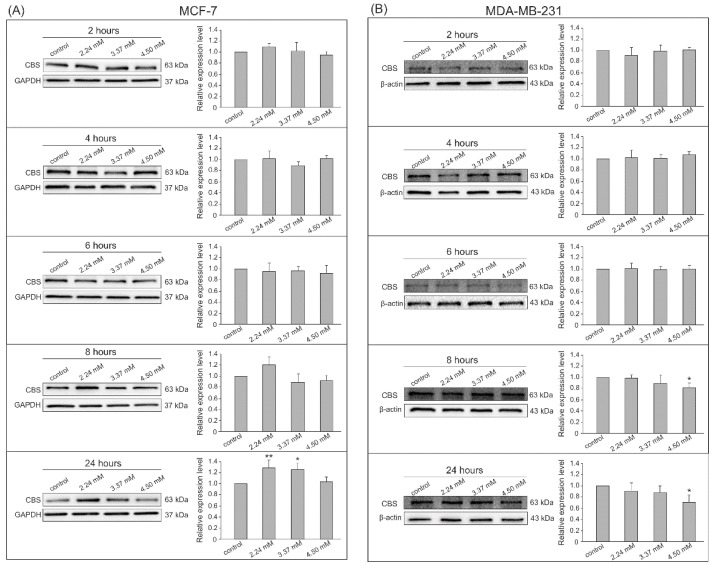
CBS expression by Western blot analysis with chemiluminescent detection and densitometric evaluation of the bands. (**A**) MCF-7 adenocarcinoma cells after 2, 4, 6, 8 and 24 h incubation with 2.24 mM, 3.37 mM, and 4.50 mM SAC concentrations, (**B**) MDA-MB-231 adenocarcinoma cells after 2, 4, 6, 8, and 24 h incubation with 2.24 mM, 3.37 mM, and 4.50 mM SAC concentrations. * *p* < 0.05, ** *p* < 0.01 SAC-treated cells vs. control. Results show representative Western blot images and densitometric evaluation of the bands from all experiments.

The Western blot analysis with chemiluminescent detection was chosen to confirm the expression of the analyzed enzymes at the protein level. The densitometry analysis was performed on changes in the expression of visualized bands from all experiments in both cell lines for MPST (Figure 3), CTH (Figure 4), and CBS (Figure 5). The conducted experiments showed that the expression of the tested sulfur enzymes at the protein level changes in adenocarcinoma cells under the influence of S-allyl-L-cysteine.

SAC contributed to an increase in MPST expression after 8 and 24 h incubation in both tested cell lines (Figure 3A,B). We confirmed a statistically significant increase in densitometric measurements for the relative bands’ intensity for the MPST after the 8 h incubation with 2.24 mM and 24 h incubation for 2.24 mM and 3.37 mM SAC in the MCF-7 cell line (Figure 3A). The 8 and 24 h incubations with all SAC concentrations significantly increased the MPST expression in the MDA-MB-231 cell line (Figure 3B).

A statistically significant change in CTH expression at the protein level in MCF-7 cells was registered only after 24 h incubation with the highest (4.50 mM) dose of SAC—the CTH expression was decreased (Figure 4A). On the other hand, MDA-MB-231 cells presented a statistically significant increase in CTH expression after 8 and 24 h of incubation with the factor regardless of its concentration (Figure 4B).

In Figure 5A, the result shows a clear trend of increasing CBS expression in the MCF-7 cell line after 24 h incubation with 2.24 mM and 3.37 mM SAC. Interestingly, incubation with the highest SAC concentration resulted in a decrease in CBS expression in MDA-MB-231 cells at 8 and 24 h but a significant difference was recorded only for the highest dose tested (Figure 5B).

## 4. Discussion

In recent years, our research investigations have focused on the presence and role of enzymes (MPST, CTH, CBS) involved in the metabolism of low-molecular-weight sulfur compounds and H_2_S in normal [31,32] and/or cancer cells [33,34]. Our other studies on an in vivo animal model confirmed the significant role of MPST and CTH in the production of hydrogen sulfide in the heart and kidneys, respectively [35]. Enzymes containing cysteine residues in the active site play a crucial role in biological processes, such as the regulation of cell cycle progression, apoptosis or signal transduction [36]. We described the CBS important role in the hydrogen sulfide metabolism in the adenocarcinoma MCF-7 cell line. We also investigated the effect of SAC on this cell line mainly regarding the induction of apoptosis. Within the previous work [25] we explored the effect of SAC on the activity and expression of enzymes involved in H_2_S production. After 24 and 48 h incubation with 2.24 mM SAC, induction of late apoptosis was observed. SAC had no significant cytotoxic effect on the MCF-7 cells upon all analyzed concentrations up to 2.24 mM SAC and had effect on decreasing cell viability. MPST, CTH, and CBS expression (at the protein level) was confirmed in non-treated MCF-7 cells as well as normal breast cells MCF-12A. There was a significant decrease in MPST activity at 2.24 mM SAC after 24 h incubation [25].

The expression profile including estrogen and progesterone receptors presence (ER+/PR+), HER2 protein overexpression (HER2+) and triple-negative characterization are features categorizing the breast cancer cells into several molecular subtypes. Triple-negative breast cancer poses many therapeutical difficulties due to the ineffective hormonal therapy regiments (because of lack of estrogen or progesterone receptors), no possibility of anti-HER2 therapy, radioresistance and often resistance to available chemotherapy treatment. Thus, a need for search of effective, non-toxic anticancer agents against this type of cancer seems justified and necessary. This subtype of breast cancer (ER-/PR-, HER2-) is represented in MDA-MB-231 cells [37,38,39,40].

All of the above information resulted in the present manuscript and the decision to further investigate the SAC effect on proliferation and expression of the MPST, CTH, and CBS enzymes with broadened cancer model. The choice of two adenocarcinoma cell lines was made in accordance with our extended previous research regarding the MCF-7 cell line [25] and with the careful consideration of the literature regarding the MDA-MD-231 as a representant of the triple-negative breast cancer, characterized as the most malignant and the most difficult to treat [41,42], as well as one being potent to SAC exposure. In research from 2008, Gapter et al. [43] presented the results regarding sub-lethal SAC-treatment as one altering mammary tumor cell adhesion and invasion through components of the extracellular matrix in MDA-MB-231 cells. Our results indicate the possible antiproliferative properties of SAC on both types of breast adenocarcinoma, in a dose and time-dependent manner. A significant decrease in viable cells was registered for whole incubation time panel in MCF-7 cells with the highest (4.50 mM) SAC concentration. In the case of the MDA-MB-231 cell line, the statistically significant antiproliferative effect was obtained by incubating the cells with SAC at a concentration of 4.50 mM for 6 and 24 h only. Considering the results of the experiments presented in the manuscript regarding the proliferation aspect in particular, the MCF-7 cell line appears to be a more sensitive one to SAC exposure. Triple-negative MDA-MB-231 cell line appears to be much more SAC-resistant regarding the reduction of viable cells number. Similar findings regarding antiproliferative properties in various cancer cell and animal models were described for other naturally occurring sulfur compound, S-adenosyl-L-methionine. In gastric and colon cancer cell models, it showed cell growth inhibition in cancer cells [44]. A well described feature of N-acetyl-L-cysteine (NAC)—another sulfur compound—was its ability to inhibit proliferation. The antiproliferative activity of NAC in the SH-SY5Y cells was associated with an increase in the MPST activity and intracellular level of sulfane sulfur [45]. Among others, SAC was described to prevent cells growth, suppress invasive growth, and inhibit proliferation in the LA-N-5 human neuroblastoma cell line [46], prostate, ovarian nasopharyngeal and esophageal cancer cell lines [47], as well as hepatocellular carcinoma MHCC97 cell model [48]. The inhibition in proliferation was also proven for other compounds in the MCF-7 cell line model [49], while MDA-MB-231 was described as one not responsive to the antiproliferative agents. In the study by Abbasi Gamasaee et al. [50], the MDA-MB-231 was compared with another breast cancer cell line MDA-MB-175-VII upon hypericin exposure, and the measurement of cell death was performed by MTT assay. The MDA-MB-231 was proved to be less responsive to treatment. In the research conducted by Liu et al. [51], the organoantimony (III) compound decreased the viability of both MDA-MB-231 and MCF-7, causing necrotic cell death in MDA-MB-231, as well as LDH release in a time- and dose-dependent manner. Differently, the studies comparing the cytotoxic effect of verbascoside conducted on MCF-7 and MDA-MB-231 showed that 100 μM verbascoside has the highest cytotoxic effect on MDA-MB-231 cells [52]. Anticancer effects of a palladium (II) complex were tested against both mentioned human breast cancer cell lines, with the Pd complex strong anti-growth effect in a dose- and time-dependent manner in vitro in MCF-7 and MDA-MB-231 [53]. Both cell lines investigated in this paper were tested in the presence of other well researched sulfur-compound sulforaphane (SFN). MCF-7 as well as MDA-MB-231 were described to be responsive to treatment of SFN, by inducing apoptosis, autophagy and antiproliferative responses [54,55]. Phenethyl isothiocyanate derived from cruciferous vegetables showing the antitumor effects was described in Zhang et al. studies [56] to be reducing breast cancer stem cell-like properties by epigenetic reactivation of CDH1. In other studies, the MCF-7 and MDA-MB-231 cell lines were investigated upon the exposure of sulfasalazine acting synergistically with naturally occurring compounds from edible wild plant 3,6-Epidioxy-1,10-bisaboladiene. The combination was concluded to effectively induce cell death and was described as anticancer drug candidate [57]. In Schörder et al.’s studies [58], sulfonamide DK-164 was described to be cytotoxic in both the MCF-7 and MDA-MB-231 cell lines. Finally, S-allyl mercaptocysteine (SAMC)—an S-oxide of S-allyl-cysteine, was found to exert multi-antitumor activities. SAMC exhibited an effective cell growth inhibition of both MCF-7 and MDA-MB-231 in a dose- and time-dependent manner by inducing cell cycle arrest in G0/G1 phase accompanied by apoptosis promotion [59]. The described effect and antitumor potential of SAC is unique for cancer cell models. Yang et al. [60] studied the effects of ABGE, the water-soluble components of mature black garlic extract that is S-allyl cysteine (SAC) and S-allyl mercaptocysteine (SMAC). In the normal mammary cell line MCF-10A, they observed that ABGE had no inhibitory effect on the growth of the cells. In other studies, plumbagin S-allyl cysteine (SAC) ester induced apoptosis in the breast cancer cell line (MCF-7) via oxidative stress, however, did not show toxicity towards the normal Vero cell line [61]. The results reported by other research teams, working with the selected cell model, correlate with our results, where for the MCF-7 line in response to the tested agent—SAC—we observe a reduction in cell viability in a dose- and time-dependent manner with simultaneous but quite moderate effect on reducing the number of viable cells in the MDA-MB-231 line.

Given the weaker response in the account of lowering the number of viable cells for the triple-negative adenocarcinoma line, the results illustrating the expression level of the tested sulfur metabolism enzymes seem very intriguing, particularly for the MPST gene, where the outcomes for both cell lines are very comparable. The results are far more interesting as for the lower concentrations in our previous research (starting from 800 μM) we did not observe statistically significant changes in MPST expression after the 24 and 48 h SAC incubations [25]. MPST activity was described to be inhibited by oxidative stress, while activated by reducing conditions [62]. It was reported that this sulfurtransferase, due to the presence of free thiol -SH groups, shows local antioxidant activity [18,19]. As such, elevated levels for the MPST enzyme in our research may be considered as a positive potential response against breast cancer cells. The antioxidant properties of SAC can be manifested by increasing the MPST expression (at the protein level).

Regarding the results for the CBS and CTH genes in the MCF-7 cell line, they are mostly consistent with our previous results for lower tested concentration of SAC. We did not observe statistically significant changes in CBS expression after the 24 and 48 h incubations (800–2245 μM SAC) in our previous research. The changes were registered for CTH, for the highest concentrations of the SAC used. Thus, it was concluded that incubation with SAC can affect CTH expression [25]. Here, we also observed the lower expression for the CTH gene for the 24 h incubation with the highest (4.50 mM) SAC concentration. Cystathionine-β-synthase and cystathionine-γ-lyase primary physiological role is to catalyze homocysteine metabolism via the transsulfuration pathway and responsible for hydrogen sulfide production involving desulfuration reactions [63]. Wang et al. [64] showed that increased CTH expression levels appeared to promote human breast cancer metastasis through the VEGF signaling pathway, and the novel CTH inhibitor I157172 suppressed the metastasis of early metastatic breast cancer cells by downregulating the VEGF signaling pathway. Since elevated CTH promotes cancer metastasis, in our experiments, the reduced expression level of this enzyme may indicate that SAC counteracts tumor propagation. This can be described as a beneficial effect towards adenocarcinoma.

As measured in the MDA-MB-231 line, there is significant increase in the CTH expression at the protein level for 8 and 24 h incubation with all SAC concentrations. The result is accompanied by a slightly decreased expression of CBS in this line for the higher concentration of SAC for the same incubation times. The overall results are interesting as they are the opposite of what we observe for the tested cell line MCF-7. The most noteworthy observed result, never registered in our previous research models, is the tendency of SAC to lower the CBS expression at the protein level. CBS is strongly overexpressed in breast cancer cells vs. the normal ones [65,66,67]. CBS involvement (indirect via the produced H_2_S) was also described as one responsible for the protective features for breast cancer against the macrophages. Cell growth was inhibited after silencing the CBS gene [65]. Taking into consideration the counsel featured in the paper by Zuhra et al. [68] that it is often difficult to determine the observed biological effects related to compounds acting as a “CBS inhibitors”, we conclude that in the triple-negative MDA-MB-231 cell line SAC may reduce the CBS expression, accompanied with the decrease in the cell growth, but further tests confirming this tendency are required to conclude the meaning of this change (such as testing the ability to generate H_2_S, cystathionine levels measurements, and determining CBS activity). Nevertheless, it seems that while overexpression of CBS is a feature that characterizes the cancer cells [66,69,70], it is a good prognosis that we observe the lowering of its expression upon S-allyl-L-cysteine exposure, and it may suggest beneficial SAC involvement against the triple-negative breast cancer cells. However, simultaneously we observe the elevated expression for CTH, that would describe quite the opposite prognosis. In Nandi et al.’s studies [71], the authors tested cardiomyocytes upon exposure of homocysteine and Na_2_S/GYY4137 (H_2_S donors) and found that homocysteine upregulates CTH but downregulates CBS, whereas Na_2_S/GYY4137 downregulates CTH but upregulates CBS in a dose-dependent manner. As such, a further detailed test regarding the adenocarcinoma model is required to conclude about the observed tendency. Metabolism of sulfur amino acids and the activity of enzymes involved in it are associated with the cell’s redox state, downstream signaling and cell proliferation. CTH is the enzyme involved for the transfer of sulfur from homocysteine to serine which results in L-cysteine, a substrate for GSH synthesis [19]. Opposite effects in the level of CTH under the influence of SAC in the cell lines studied are most likely related to the greater aggressiveness of the MDA-MB-231 cell line model. The lack of regulation by hormones provides increased levels of CTH, that will result in the formation of greater amounts of L-cysteine and GSH and a stronger antioxidant response, desirable for the MDA-MB-231 line. This is a promising observation for the presented study; however, it is an educated guess which requires deeper analysis and additional investigation combined with comparison of metabolite levels involved in sulfur compound metabolism (e.g., cysteine, GSH).

Considering the experimental results and the literature data on MCF-7, it appears to be a more sensitive line to the effects of SAC, particularly in reducing the number of viable cells. The effects associated with changes in protein levels tested by Western Blot are quite interestingly different, as in the present manuscript, we reported similar changes in the MSPT levels and quite opposite effects regarding CTH and CBS in both cell lines. The indication of changes, seen especially for the effect induced on the triple-negative MDA-MB-231, is the most interesting finding that is worth considering in further, more detailed investigations. As for now, without the further analysis of, e.g., hydrogen sulfide generation, the activity of enzymes combined with the cell’s redox state and programmed cell death, the final determination of the broader response of one of the lines and the effectiveness of SAC is still to be confirmed/discovered.

## 5. Conclusions

In this research, we confirmed the antiproliferative potential of S-allyl-L-cysteine efficient in MCF-7 and the moderate potential in MDA-MB-231 breast cancer cell lines. Our studies provided new information about the increase in MPST enzyme expression in response to S-allyl-L-cysteine in the adenocarcinoma in vitro cellular model for both the investigated cell lines. We also registered quite the opposite tendency in the expression of CBS and CTH in two investigated cell lines. With further investigation of the triple-negative model, we may be able to add some interesting knowledge to sulfur enzyme metabolism regarding breast adenocarcinoma and the regulating its activity and expression (gene silencing) in anticancer therapy.

## Data Availability

Raw data supporting the reported results can be obtained by demand from the corresponding author.

## Supplementary Materials

The following supporting information can be downloaded at: https://www.mdpi.com/article/10.3390/biom14020188/s1, Table S1: The cytotoxicity effect of S-Allyl-L-cysteine (SAC) in concentrations 2.24 mM, 3.37 mM, and 4.50 mM in the human breast adenocarcinoma cell line MCF-7 after 2-, 4-, 6-, 8-, and 24-hour incubations; Table S2: The cytotoxicity effect of S-Allyl-L-cysteine (SAC) in concentrations 2.24 mM, 3.37 mM, and 4.50 mM in the human breast adenocarcinoma cell line MDA-MB-231 after 2-, 4-, 6-, 8-, and 24-hour incubations.

## Abbreviations

ATCC—American Type Culture Collections, CBS—cystathionine β-synthase, CTH—cystathionine γ-lyase, GAPDH—glyceraldehyde-3-phosphate dehydrogenase, HER—epidermal growth factor receptor 2, LDH—lactate dehydrogenase, MPST—3-mercaptopyruvate sulfurtransferase, MTS—3-(4,5-dimethylthiazol-2-yl)-5-(3-carboxymethoxyphenyl)-2-(4-sulfophenyl)-2H-tetrazolium, PLP—pyridoxal phosphate, PVDF—polyvinylidene fluoride, SAC—S-allyl-L-cysteine, SAMC—S-allyl mercaptocysteine, SD—standard deviations, SPC—S-propyl-L-cysteine, and SPRC—S-propargyl-L-cysteine.
