# Supplementary material for: S-Allyl-L-Cysteine Affects Cell Proliferation and Expression of H2S-Synthetizing Enzymes in MCF-7 and MDA-MB-231 Adenocarcinoma Cell Lines
Source: Biomolecules. 2024 Feb 4;14(2):188. doi: 10.3390/biom14020188 (PMC10886539; doi:10.3390/biom14020188)
Supplement: Supplementary file 1 [file biomolecules-14-00188-s001.zip › biomolecules-2821198-supplementary.pdf]

Tables S1 and S2 show the **c**ellular plasma membrane integrity **c**hanges measured by the LDH leakage in MCF-7 and MDA-MB-231 accordingly. The obtained data indicate no cytotoxic effect on MCF-7 and MDA-MB-231 adenocarcinoma cell lines upon the SAC exposure. For both tested lines in tested timeline upon chosen concentrations there were no detectible LDH activity, except the one value exceeding 0 recorded for 24 h incubation with 2.24 mM SAC concentration, at a level lover than 1% confirming no cytotoxic effect of the compound.

Table S1. The cytotoxicity effect of S-Allyl-L-cysteine (SAC) in concentrations 2.24 mM, 3.37 mM, and 4.50 mM in the human breast adenocarcinoma cell line **MCF-7** after 2-, 4-, 6-, 8-, and 24-hour incubations.

**SAC**	**% of cytotoxicity**				
	**2 h**	**4 h**	**6 h**	**8 h**	**24 h**
**2.24 mM**	0	0	0	0	0.9
**3.37 mM**	0	0	0	0	0
**4.50 mM**	0	0	0	0	0

Table S2. The cytotoxicity effect of S-Allyl-L-cysteine (SAC) in concentrations 2.24 mM, 3.37 mM, and 4.50 mM in the human breast adenocarcinoma cell line **MDA-MB-231** after 2-, 4-, 6-, 8-, and 24-hour incubations.

**SAC**	**% of cytotoxicity**				
	**2 h**	**4 h**	**6 h**	**8 h**	**24 h**
**2.24 mM**	0	0	0	0	0
**3.37 mM**	0	0	0	0	0
**4.50 mM**	0	0	0	0	0
