# Supplementary material for: S-Allyl-L-Cysteine Affects Cell Proliferation and Expression of H2S-Synthetizing Enzymes in MCF-7 and MDA-MB-231 Adenocarcinoma Cell Lines
Source: Biomolecules. 2024 Feb 4;14(2):188. doi: 10.3390/biom14020188 (PMC10886539; doi:10.3390/biom14020188)
Supplement: Supplementary file 1 [file biomolecules-14-00188-s001.zip › Original Western blot pictures of Figure 4A.zip!/Figure 4a/4:6 CTH 4h_and_6h_Mcf7.pdf]

Image Report: milab 2021-03-23_11h03m52s

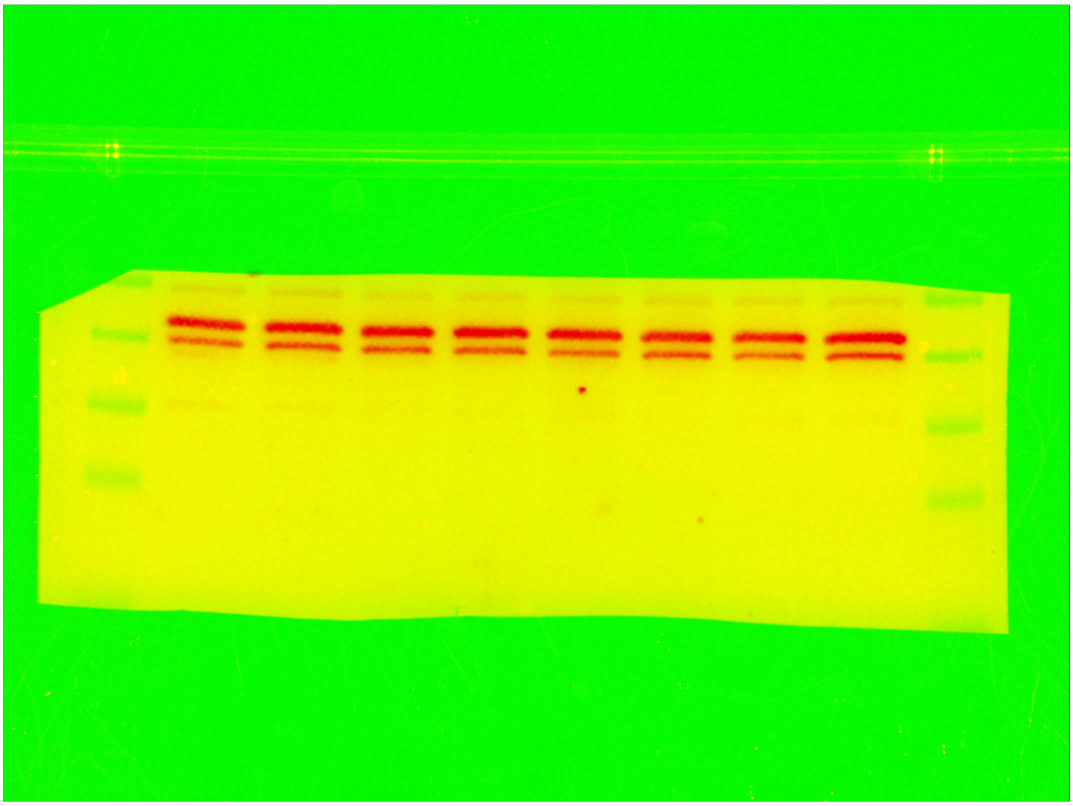

Image Information

Imager	ChemiDoc MP
Serial Number	731BR02754
Software Version	6.1.0.07
Acquisition Date	23/3/2021 11:04:13 AM
User Name	milab
Image Area (mm)	X: 95.0 Y: 71.0
Pixel Size (µm)	X: 68.2 Y: 68.2

Channel 1 - Red - Colorimetric

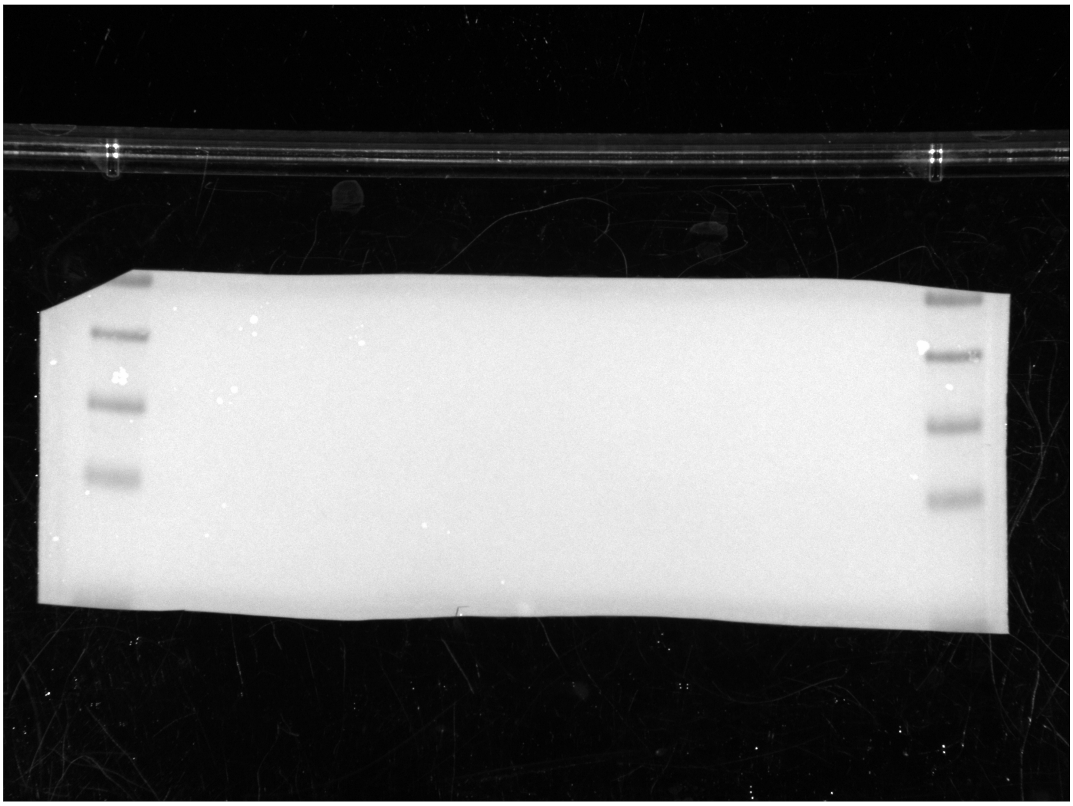

Acquisition Information

Exposure Time (sec)	0.053 (Auto - Intense Bands)
Flat Field	Applied (Lens)
Application	Colorimetric
Channel Color	Red
Excitation Source	White Epi Illumination
Emission Filter	Standard Filter
Data Range (Int)	0 - 65251

Analysis Settings

No analysis performed

Channel 2 - Green - Chemi Hi Sensitivity

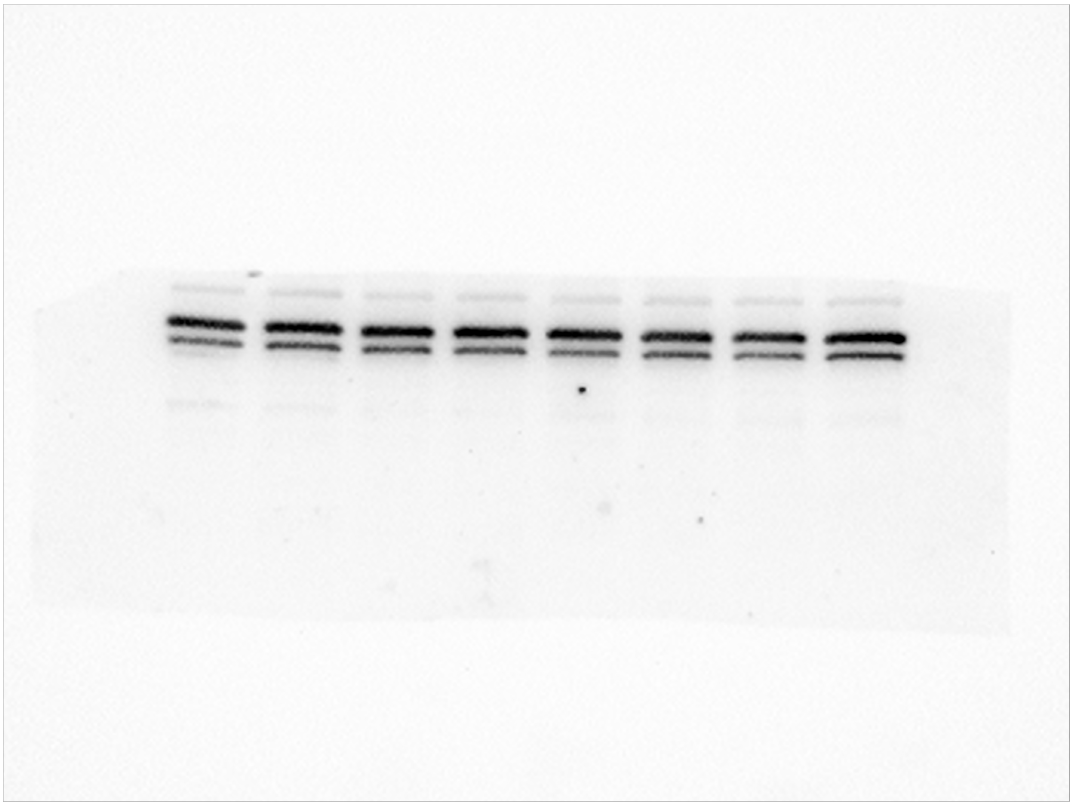

Acquisition Information

Exposure Time (sec)	33.416 (Auto - Intense Bands)
Flat Field	Applied (Lens)
Application	Chemi Hi Sensitivity
Channel Color	Green
Excitation Source	No Illumination
Emission Filter	No Filter
Binning	4x4
Data Range (Int)	92 - 65534

Analysis Settings

No analysis performed
