# Supplementary material for: S-Allyl-L-Cysteine Affects Cell Proliferation and Expression of H2S-Synthetizing Enzymes in MCF-7 and MDA-MB-231 Adenocarcinoma Cell Lines
Source: Biomolecules. 2024 Feb 4;14(2):188. doi: 10.3390/biom14020188 (PMC10886539; doi:10.3390/biom14020188)
Supplement: Supplementary file 1 [file biomolecules-14-00188-s001.zip › Original Western blot pictures of Figure 4B.zip!/Figure 4b/2 CTH not-shown_and_2h_MDA_MB.pdf]

Image Report: chemidoc 2022-02-09_13h23m52s

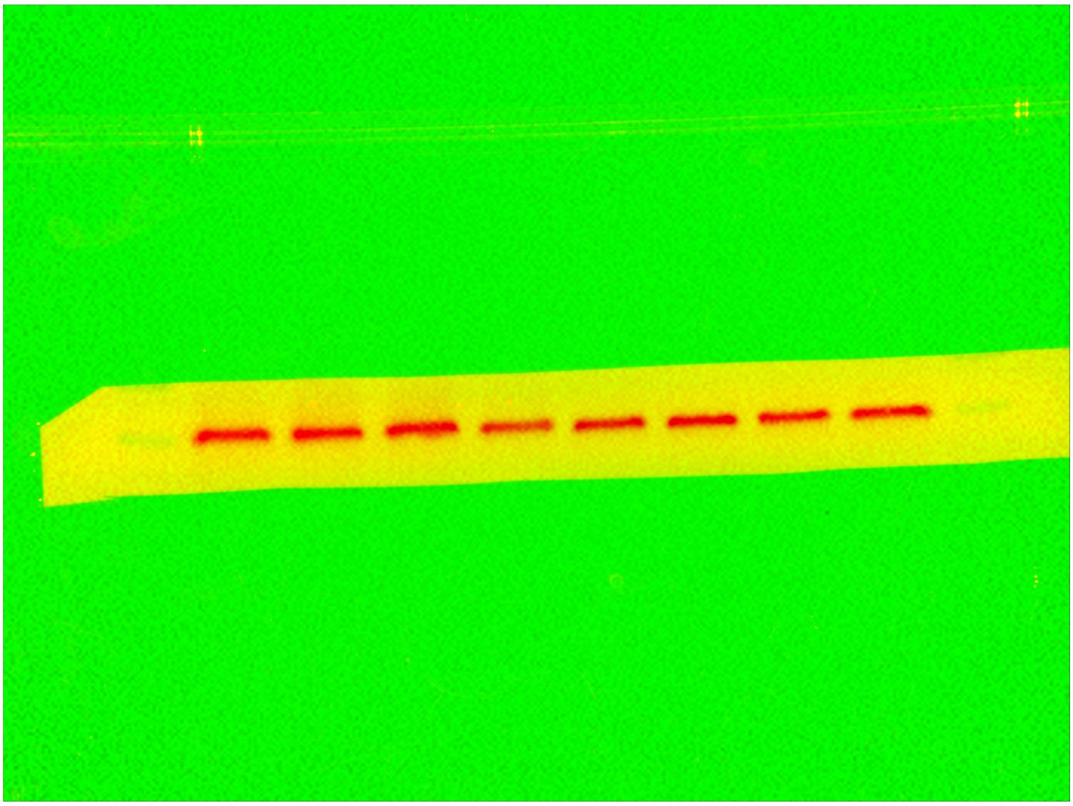

Image Information

Imager	ChemiDoc MP
Serial Number	731BR02754
Software Version	6.1.0.07
Acquisition Date	9/2/2022 1:24:11 PM
User Name	chemidoc
Image Area (mm)	X: 95.0 Y: 71.0
Pixel Size (µm)	X: 68.2 Y: 68.2

Channel 1 - Red - Colorimetric

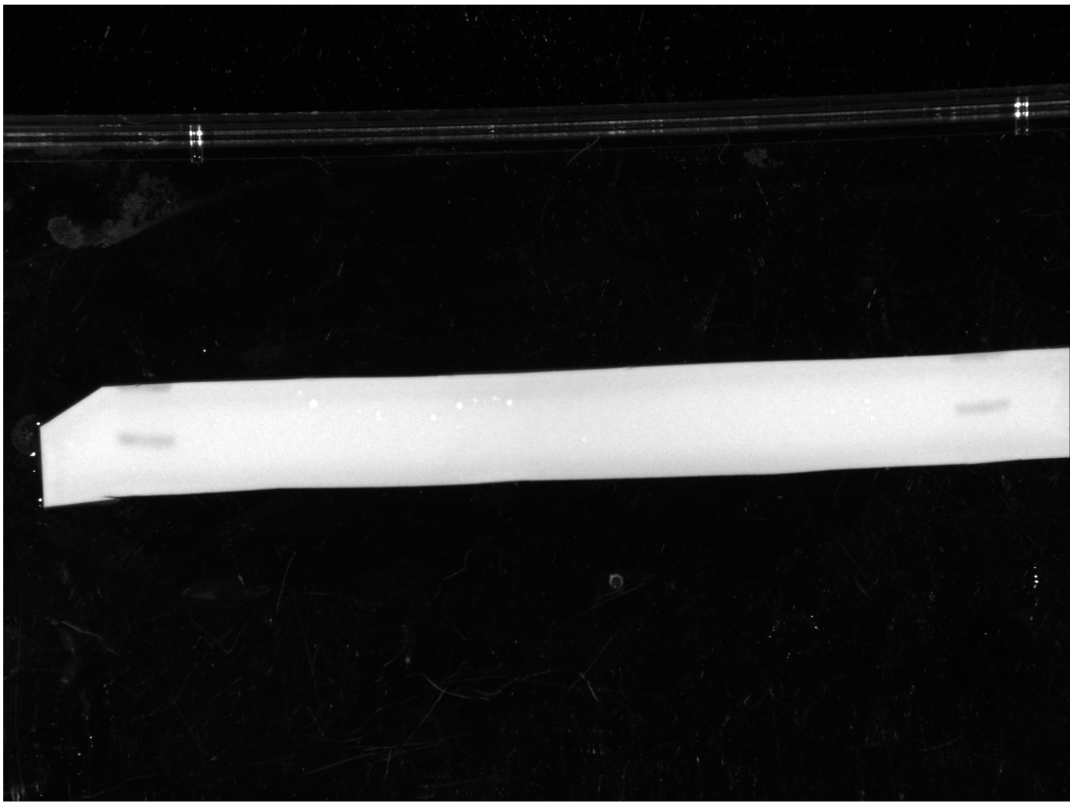

Acquisition Information

Exposure Time (sec)	0.055 (Auto - Intense Bands)
Flat Field	Applied (Lens)
Application	Colorimetric
Channel Color	Red
Excitation Source	White Epi Illumination
Emission Filter	Standard Filter
Data Range (Int)	0 - 65455

Analysis Settings

No analysis performed

Channel 2 - Green - Chemi Hi Sensitivity

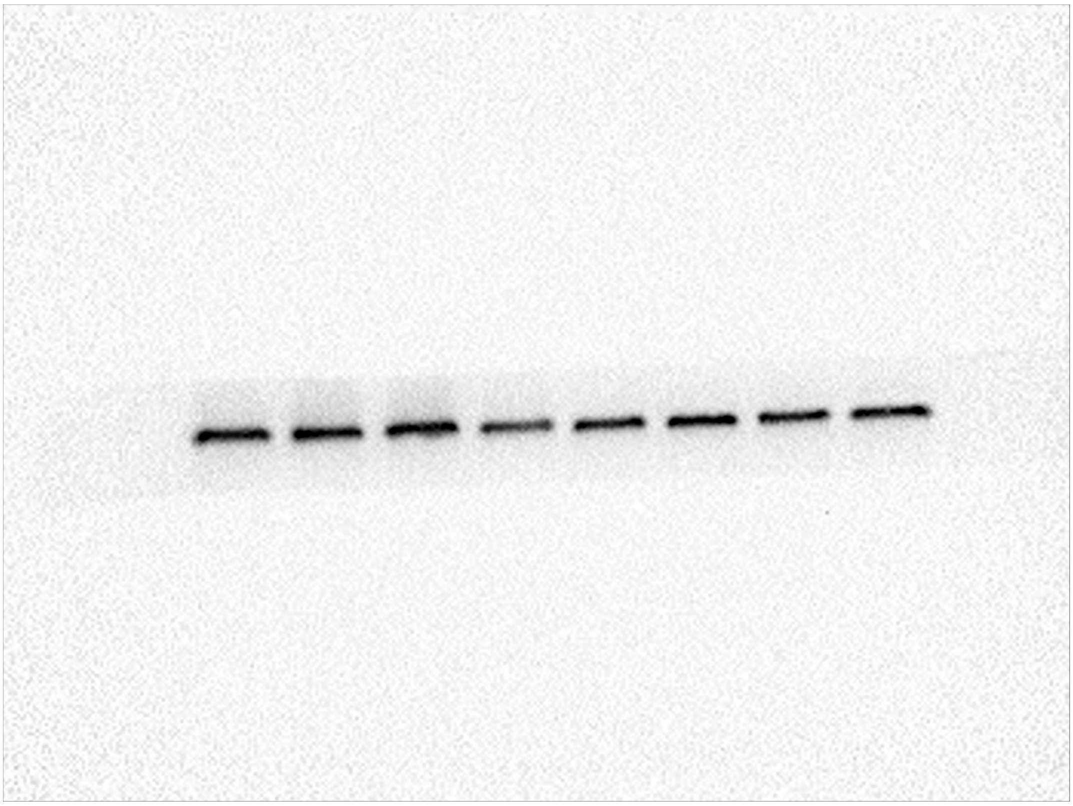

Acquisition Information

Exposure Time (sec)	16.166 (Signal Accumulation)
Flat Field	Applied (Lens)
Application	Chemi Hi Sensitivity
Channel Color	Green
Excitation Source	No Illumination
Emission Filter	No Filter
Binning	4x4
Data Range (Int)	0 - 16436

Analysis Settings

No analysis performed
