# Supplementary material for: S-Allyl-L-Cysteine Affects Cell Proliferation and Expression of H2S-Synthetizing Enzymes in MCF-7 and MDA-MB-231 Adenocarcinoma Cell Lines
Source: Biomolecules. 2024 Feb 4;14(2):188. doi: 10.3390/biom14020188 (PMC10886539; doi:10.3390/biom14020188)
Supplement: Supplementary file 1 [file biomolecules-14-00188-s001.zip › Original Western blot pictures of Figure 4B.zip!/Figure 4b/6 CTH not-shown_and_6h_MDA_MB.pdf]

Image Report: chemidoc 2022-02-09_13h29m04s

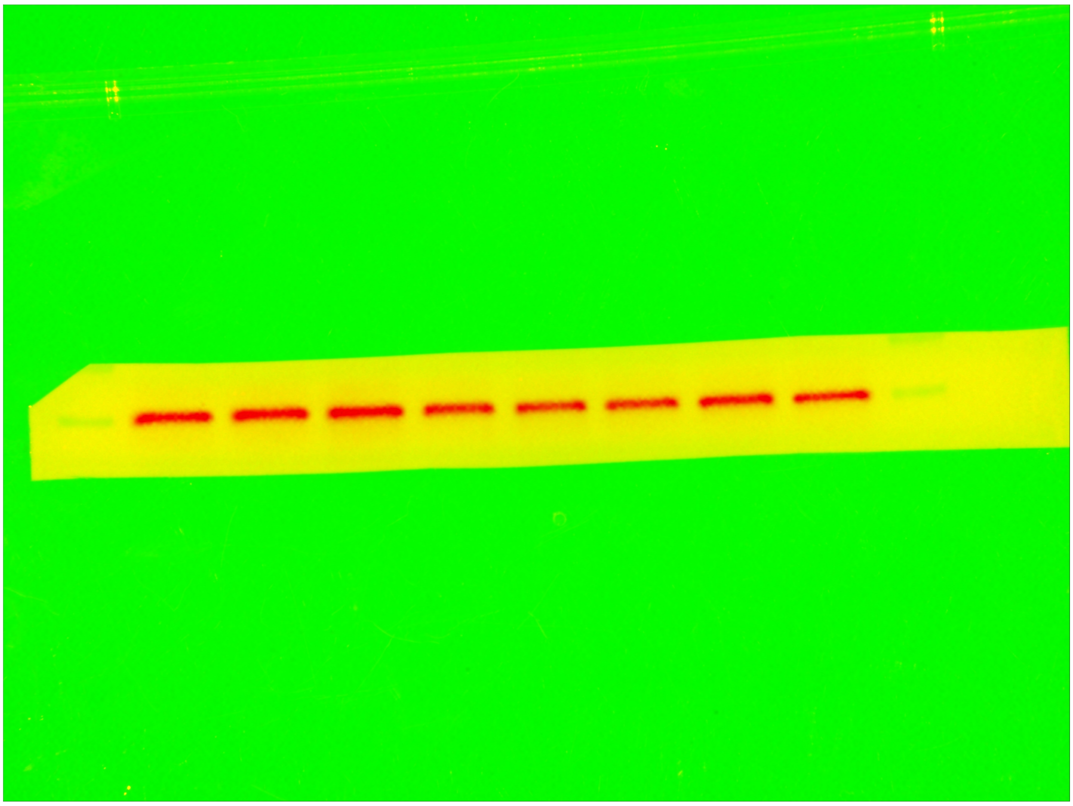

Image Information

Imager	ChemiDoc MP
Serial Number	731BR02754
Software Version	6.1.0.07
Acquisition Date	9/2/2022 1:29:25 PM
User Name	chemidoc
Image Area (mm)	X: 95.0 Y: 71.0
Pixel Size (μm)	X: 68.2 Y: 68.2

Channel 1 - Red - Colorimetric

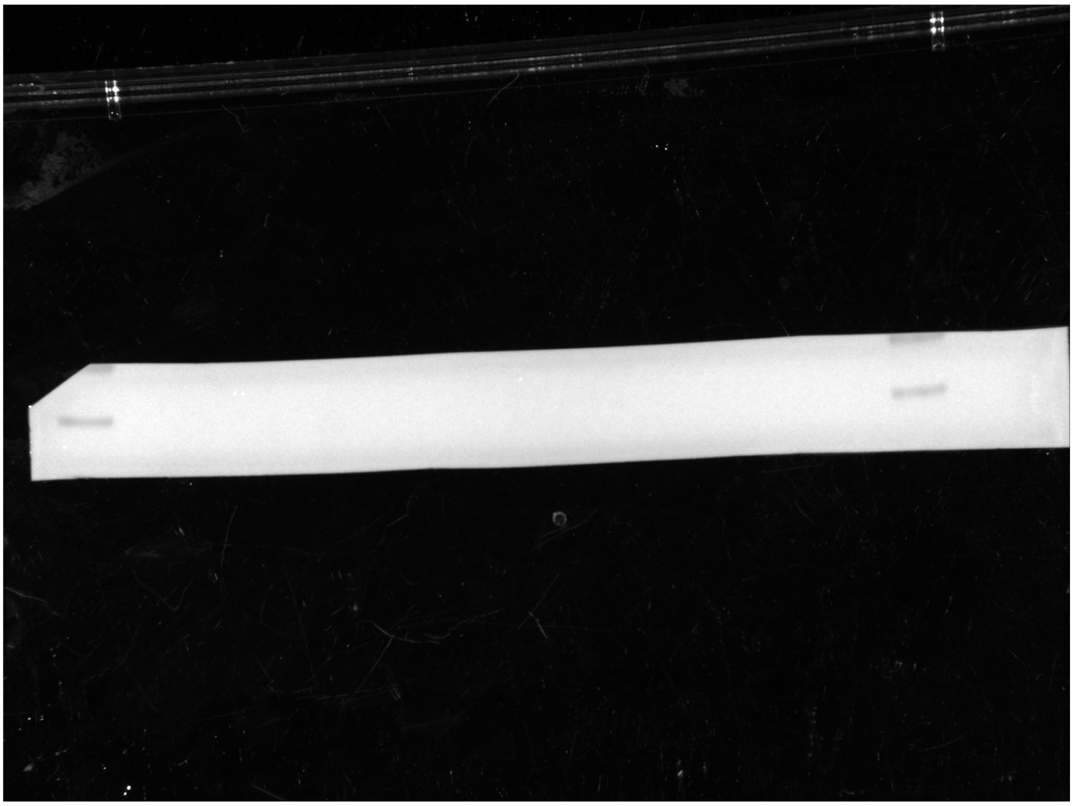

Acquisition Information

Exposure Time (sec)	0.054 (Auto - Intense Bands)
Flat Field	Applied (Lens)
Application	Colorimetric
Channel Color	Red
Excitation Source	White Epi Illumination
Emission Filter	Standard Filter
Data Range (Int)	0 - 65335

Analysis Settings

No analysis performed

Channel 2 - Green - Chemi Hi Sensitivity

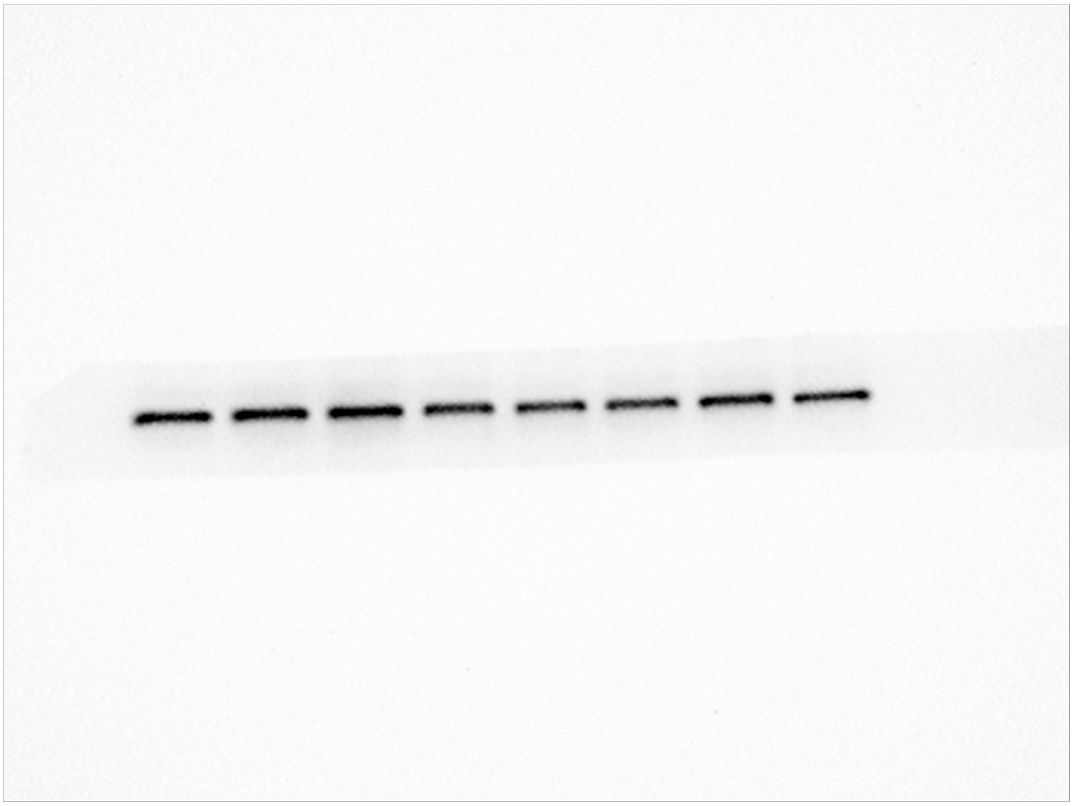

Acquisition Information

Exposure Time (sec)	57.229 (Auto - Intense Bands)
Flat Field	Applied (Lens)
Application	Chemi Hi Sensitivity
Channel Color	Green
Excitation Source	No Illumination
Emission Filter	No Filter
Binning	4x4
Data Range (Int)	0 - 65534

Analysis Settings

No analysis performed
